# Unintended consequences of existential quantifications in biomedical ontologies

**DOI:** 10.1186/1471-2105-12-456

**Published:** 2011-11-24

**Authors:** Martin Boeker, Ilinca Tudose, Janna Hastings, Daniel Schober, Stefan Schulz

**Affiliations:** 1 Institute of Medical Biometry and Medical Informatics, University Medical Center Freiburg, Germany; 2 Chemoinformatics and Metabolism, European Bioinformatics Institute, Hinxton, UK; 3 Institute for Medical Informatics, Statistics and Documentation, Medical University of Graz, Austria

## Abstract

**Background:**

The Open Biomedical Ontologies (OBO) Foundry is a collection of freely available ontologically structured controlled vocabularies in the biomedical domain. Most of them are disseminated via both the OBO Flatfile Format and the semantic web format Web Ontology Language (OWL), which draws upon formal logic. Based on the interpretations underlying OWL description logics (OWL-DL) semantics, we scrutinize the OWL-DL releases of OBO ontologies to assess whether their logical axioms correspond to the meaning intended by their authors.

**Results:**

We analyzed ontologies and ontology cross products available via the OBO Foundry site http://www.obofoundry.org for existential restrictions (*someValuesFrom*), from which we examined a random sample of 2,836 clauses.

According to a rating done by four experts, 23% of all existential restrictions in OBO Foundry candidate ontologies are suspicious (Cohens' *κ *= 0.78). We found a smaller proportion of existential restrictions in OBO Foundry cross products are suspicious, but in this case an accurate quantitative judgment is not possible due to a low inter-rater agreement (*κ *= 0.07). We identified several typical modeling problems, for which satisfactory ontology design patterns based on OWL-DL were proposed. We further describe several usability issues with OBO ontologies, including the lack of ontological commitment for several common terms, and the proliferation of domain-specific relations.

**Conclusions:**

The current OWL releases of OBO Foundry (and Foundry candidate) ontologies contain numerous assertions which do not properly describe the underlying biological reality, or are ambiguous and difficult to interpret. The solution is a better anchoring in upper ontologies and a restriction to relatively few, well defined relation types with given domain and range constraints.

## Background

OBO, the Open Biomedical Ontologies project, originated as a collection of controlled vocabularies [1]. At that time, OBO ontologies consisted of terms, which were interconnected by typed binary relationships, such as *is*_*a *and *part_of*. Since then, OBO's scope was augmented towards medicine and it was therefore renamed from "Open Biological Ontologies" to "Open Bio*medical *Ontologies". It was supplemented by a formal language, the OBO file format, which grew in semantic complexity over time. The use of the Semantic Web standard ontology language OWL [2,3] based on description logics (DLs) [4], was encouraged, and tools for conversion between the OBO file format and OWL were proposed [5]. Finally, a set of principles was proposed for the coordinated development of non-overlapping ontologies [6].

The OBO Site provides three different kinds of ontologies (all ontologies referred to in this paper are available via the OBO Foundry portal http://www.obofoundry.org). *We therefore refrain from indicating references to specific ontologies mentioned in the paper*.:

• The *OBO Foundry ontologies*, a selection of eight ontologies that, after expert review performed in 2010, were declared to sufficiently comply with the OBO Foundry principles. The following ontologies constitute the OBO Foundry collection: (i) CHEBI: Chemical Entities of Biological Interest; (ii) GO: Gene Ontology Cellular Component; (iii) GO: Gene Ontology Molecular Function; (iv) GO: Gene Ontology Biological Process; (v) PATO: Phenotypic Quality Ontology; (vi) PRO: Protein Ontology; (vii) XAO: Xenopus Anatomy Ontology; and (viii) ZFA: Zebrafish Anatomy Ontology.

• The *OBO Foundry candidate ontologies and other ontologies of interest*. This is a heterogeneous, steadily growing collection of currently 91 ontologies, only a few of which claim to follow the OBO Foundry principles. Among this set six candidate ontologies were considered close to being included into the Foundry [7], viz.: (i) CL: Cell Ontology; (ii) FMA: Foundational Model of Anatomy Ontology; (iii) EnvO: Environment Ontology; (iv) HPO: Human Phenotype Ontology; (v) OBI: Ontology for Biomedical Investigations; and (vi) SO: Sequence Ontology.

• A collection of ontologies called 'Mappings between, logical definitions for, bridging, and relations for combining, ontologies', contains 62 resources [8]. They consist of 22 mapping files, one ontology (BFO), four bridges, four relation ontologies and 31 cross-product ontologies with logical definitions.

In the work presented here, we aim to analyze the correctness of the use of logic by the OBO Foundry or close-to OBO Foundry ontologies and related mappings. We concentrate on OWL, as this is considered the language of choice for creating and exchanging ontologies [3]. OWL subscribes to a model-theoretic semantics which leads to logically crisp and far-reaching entailments bearing the risk of creating unintended implications or misinterpretations. We use the phrase "unintended consequence" to describe assertions, and entailments from assertions, which are contrary to the intention of the modeler. Their identification and prevention is crucial for good quality, as otherwise automated reasoning produces unreliable results. The paper is organized as follows. In the next subsections of the Background section we will provide terminological clarifications and insight into OBO and OWL syntax and semantics. The Methods section describes the sampling, rating and evaluation of a key element in OWL ontologies, *existential restrictions*, which we hypothesize as constituting a major source of axioms leading to unintended consequences in biomedical ontologies. In the rather extensive Discussion section classes of erroneous modeling decisions are illustrated by examples and possible alternatives are discussed. In the concluding section we summarize the lessons learnt from this experiment and give suggestions for improving ontology quality in the OBO Foundry.

### Terminologies vs. Ontologies

Here we introduce the basic concepts underlying our work, highlighting the implications resulting from commitment to different paradigms for semantics (terminology vs. description logics) and syntax (OBO vs. OWL).

The need for standards to semantically annotate different kinds of resources has been addressed by controlled vocabularies and terminology systems [9], language-oriented artifacts that relate word senses by informal thesaurus-style relations. The need to facilitate the interpretation of these language-oriented artifacts by computers initiated a trend of formalizing their semantics, which was supported by logic-based ontologies. The Gene Ontology (GO) [10] was a pioneer for moving from a purpose-oriented annotation vocabulary to a more principled resource. GO has been one of the driving forces of OBO. It is also motivated by the evolution of ontological principles rooted in analytical philosophy, as well as by cross-fertilization between the Semantic Web [11] and Life Sciences communities [12,13].

### OBO vs. OWL

The move from the OBO format to OWL mirrors this progress from the representation of term meanings towards the representation of the domain entities that the terms denote and their properties. The Web Ontology Language OWL [2], now available in its second release [3], provides an abstract syntax for a language encompassing different flavors of description logics (DLs), a family of decidable fragments of first-order logic [4]. In contrast, the OBO flatfile format [14] represents a semantic network of nodes (terms) and edges (relationships), together with metadata and linguistic information (synonyms). At its current state, the OBO format is not a formal language. The definition of a formal semantics for the language is a work in progress. For a draft of the OBO syntax and semantics see http://berkeleybop.org/~cjm/obo2owl/obo-syntax.html. A preliminary implementation thereof is available at http://code.google.com/p/oboformat/. To further elucidate the distinction between these two formalisms, consider the following example from the mouse anatomy ontology.

This extract asserts the relationship *part*_*of *between the terms ankle and hindlimb in OBO format.

[Term]

id: MA:0000043

name: ankle

relationship: part of MA:0000026 ! hindlimb

This assertion does not commit to a semantics in terms of the real world entities which are denoted by the terms. It does not allow us to infer that, e.g., all hindlimbs have ankles, or all ankles are part of a hindlimb. Descriptions at this level require some kind of ontological interpretation for the OBO syntax in terms of OWL axioms, as OWL axioms are explicitly quantified. One such interpretation is given by the OBO2OWL specification [15]. According to this specification, each relationship in OBO format translates to the following existential OWL restriction, illustrated in the compact OWL Manchester syntax [16]:

*Ankle *subClassOf **part**_**of **some *Hindlimb*

Making proper use of description logics (and avoiding unintended consequences) requires understanding their very crisp notions of "class" and "relationship". Classes such as *Ankle *are interpreted as sets of all individuals that correspond to the definitional criteria of that class, i.e., here: all particular ankles in the domain of mouse anatomy. Relationships are then sets of pairs of class instances like **has_part **or **part_of**, which extend to all pairs of objects in the domain that are related in terms of parts and wholes. So, all pairs of mouse ankle instances with their respective *Hindlimb *instances are in the extension of the relation **part**_**of**.

It is the reference to instances that makes up the greatest difference between the OBO term-based approach and the OWL class approach, and which explains why the latter is semantically more precise. The description logics on which OWL is based, in contrast to OBO, cannot straightforwardly assert relationships directly between terms or classes. As shown above, relationships always hold between individuals and need to be quantified when classes are to be connected. Quantification can consist in existential quantification ("some", ∃), universal value restriction ("only", ∀), or cardinality restrictions (max *n*; min *n*; exactly *n*). Our mouse limb example could therefore be alternatively translated into at least the following three OWL expressions:

(i) *Ankle *subClassOf **part**_**of **some *Hindlimb*

(ii) *Ankle *subClassOf **part**_**of **exactly 1 *Hindlimb*

(iii) *Ankle *subClassOf **part**_**of **only *Hindlimb*

(i, the existential restriction) expresses that every instance of the class ankle is part of at least one instance of the class *Hindlimb*;

(ii, the cardinality restriction) is stricter and expresses that every instance of the class ankle is part of exactly one instance of the class *Hindlimb*;

(iii, the universal restriction) expresses that an instance of the class ankle can only be part of instances of the class *Hindlimb*.

In this case the choice of (i) as the default OBO to OWL translation target representation looks plausible. At least with the relation **part**_**of**, the option (ii) would be too strict, and the representation (iii) would conflict with the transitivity behavior of the relation **part**_**of**, since an instance of the class ankle is also part of the body that the hindlimb is part of.

### Ontological dependence

*Generic *(G) ontological dependence can be defined according to [17] as:

*x *depends_*G *_for its existence upon *Fs *= _*df*_

Necessarily, *x *exists only if some *F *exists

The first two representations - (i) and (ii) above - express ontological dependence between the two classes, that is, that there is no ankle without a hindlimb it is part of, by the semantics assigned to the *some *and *exactly *OWL constructs, namely, that for each instance of the first class there is at least one instance of the related class. Thus, every instance of *Ankle *existentially depends on some instance of *Hindlimb*. Representation (iii) has a remarkable property, which might be easier seen in an equivalent formulation:

(iii') *Ankle *subClassOf

not (**part**_**of **some not *Hindlimb*)

In contrast to (i) and (ii), proposition (iii) does not express any ontological dependence. Bearing in mind that the first representation is the one favored by the OBO2OWL conversion, which makes most of the native OBO ontologies available in OWL, the question is now whether its very strong claim about dependence can be upheld for each and every relational statement in OBO ontologies and cross products. There are many kinds of relational statements for which this claim is obviously too strong. We will certainly not want to interpret statements such as "Aspirin treats headache", or "Smoking causes cancer" in the sense that there is some headache for each and every aspirin tablet, or that there is no smoking event that is not a cause of some cancer.

## Methods

### Sampling

The scope of our study included the following representational artifacts for which one of the following conditions applied:

1. Official Foundry Ontologies;

2. Foundry candidates close to approval (see above);

3. Mappings that use only ontologies from 1 or 2.

A general condition is that a native or derived OWL file exists. With one exception (OBI) the ontologies under scrutiny are being developed and maintained in OBO format.

Our analyses distinguish between (i) "Foundry" and (ii) "Cross Products", with 14 (13, HPO, the human phenotype ontology does not contain any relational statements,) and 11 members, respectively. In the first group we found 65 different relation types, and in the second one 102. A sampling algorithm was defined as follows: For each relation per ontology at most 20 instances of existentially quantified relational statements were selected at random. The limitation to 20 was chosen due to the high number of relation/ontology pairs, and to the non-trivial effort involved in arriving at a conclusion which often required consultation of specific textbooks and Internet resources.

These samples were presented to the raters in the following tabular format:

*Source*_*Ontology *ontology name

*Class*_*Source*   name of the class to be described

*Relation*           name of the relation used within

                        a "someValuesFrom" statement

*Class_Target*    name of the class used within

                        a "someValuesFrom" statement

### Expert rating

The plausibility of the relational statements was assessed by four of the authors (SS, MB, JH, DS) with backgrounds in medicine, biology and chemistry and expertise in ontology engineering and theory, two of whom having practical experience in OBO ontology projects. A certain difficulty was posed by the fact that no single rater's expertise covered the entirety of the domains the selected ontologies were about. To address this shortcoming, the experts trained for the relationship rating in an earlier pilot phase using samples comparable to (but different from) those used in the final study. Disagreements were discussed in consensus sessions.

Each of the experts analyzed 946 statements. One quarter of the statements were randomly assigned to be analyzed by two experts, which enabled the computation of the inter-annotation agreement using kappa statistics. The expert judgments address the following question:

Is the ontological dependence of *Class*_*Source *on *Class*_*Target *justified? {no, yes}. More precisely: does there exist for every instance of the source class at least one instance of the target class which is related by relation R?

To properly answer this question the scope of the underlying ontology had to be considered, i.e. whether the ontology is limited to canonical entities, such as the Foundational Model of Anatomy. Where there was reasonable doubt about a rating, a positive rating is given. The adequacy of the relation type chosen was not considered for the assessment, because the meaning of many relations is not further defined. For example, the statement

*<Wall of intestine*; **constitutional**_**part**_**of**;

*Intestine*>

was rated as positive (because there are no intestine walls without intestines) even if a rater would have deemed the relation **part_of **as more adequate. The interrater agreement on categorical data was calculated according to Cohen's kappa [18].

### Analysis and revision

The erroneous statements are analyzed by the authors and categorized by error type. The reasons behind the modeling errors are investigated and alternative modelling choices are proposed.

## Results and Discussion

### Rating outcomes

**Figure 1:**
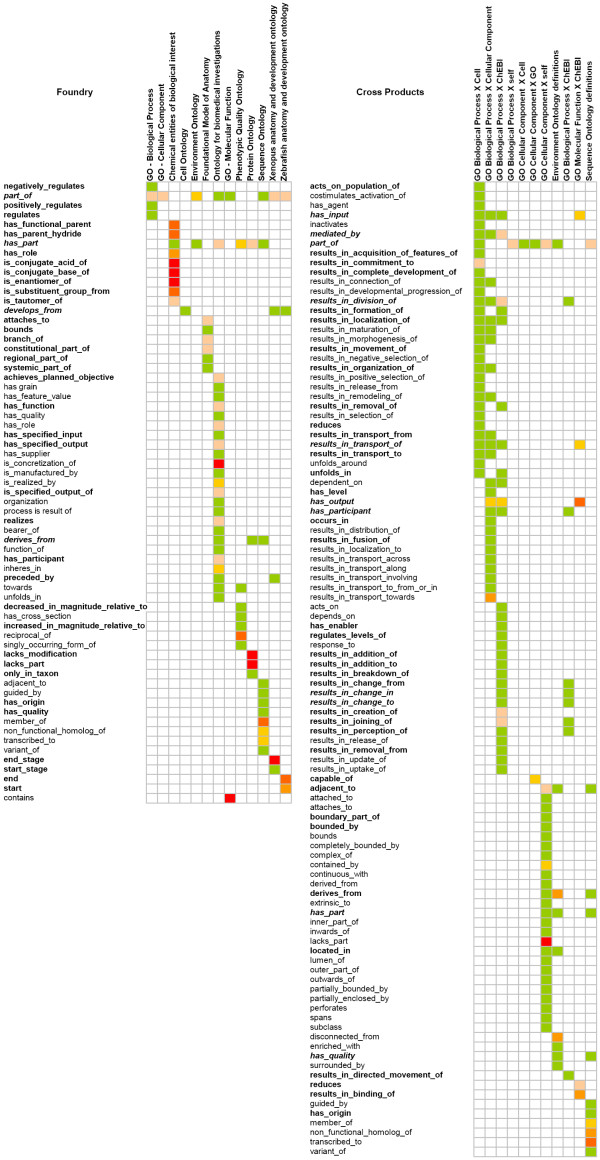
**Occurrence of existential restrictions in DL axioms**. Occurrence of existential restrictions in DL axioms in OBO Foundry ontologies and close candidates (left), cross products (right). Rating results as heat map: green - no erroneous restrictions to deep red - only erroneous restrictions. Sample size is encoded as normal font: less than 20 axioms, **boldface: 20-39 axioms **and ***italic boldface: 40 and more axioms***.

Figure 1 visualizes the distribution of relations across ontologies with colors depicting the rates of erroneous ratings. Additional files 1 and 2 present the exact results, together with estimates, for relations and ontologies respectively. Figure 1 shows (1) a high specificity of relations per ontology (only a few relations, mostly OBO relations [19] such as **part**_**of **or **derives**_**from **occur in several ontologies) and a high rate of obviously unproblematic relations, to a greater extent in the cross products than in the ontologies.

Most errors in the Foundry ontologies occurred in relations linking chemical entities, in references to end stages or outcomes of processes, and "lacks" relations. Errors in the cross products collection are mainly due to input/output relations (Additional file 1).

The estimate of false existential restrictions amounts to 17,932 [12,227; 26,018]^95% ^for the total Foundry relations (≈23%) and 2,827 [1,914; 4,191]^95% ^for the Cross Product relations (≈15%). The ontology with the highest rate of false restrictions is ChEBI with 62% rated as incorrect, which is a factor of the large number of problematic chemical relations specific to this ontology (Additional file 2). Error rates higher than 30% also characterize the Zebrafish ontology, the Environmental ontology and the Protein ontology. All Cross Products but two have relatively low error rates. The exceptions are 'GO Cellular Component X GO' (24%) and 'GO Molecular Function X ChEBI' (42%). It is remarkable that there is a significant difference in overall error rate comparing the "official" OBO Foundry ontologies to the Foundry candidates, despite the fact that the problematic relations are widely used in the cross-products.

Although the four raters discussed many controversial examples in a pilot study, the interrater agreement was only moderate. Cohen's Kappa amounted to 0.6 [0.46, 0.76]^95% ^across all ratings, with a very large difference between the two sets: For the Foundry sample the agreement was quite good (Kappa = 0.78 [0.64, 0.92]^95%^), whereas the agreement was extremely low for the Cross Product sample (Kappa = 0.07 [-0.14, 0.29]^95%^), The poor Kappa in the Cross Product sample is partly due to the prevalence dependency of the Kappa measure, as with very low or high prevalence of the binary variable, Kappa tends to be low as a direct consequence of its definition and its aim to adjust a raw agreement rate with respect to the expected amount of agreement under choice conditions [20]. The marginal prevalence of false existential restrictions was 244/1186 = 0.21 in the Foundry sample and only 83.5/1650 = 0.05 in the Cross Product sample. The low error prevalence limits the value of the crossproduct sample, but it can still be interpreted in the sense that with the exception of a few relation types the cross product sample contains less errors than the other sample.

### Error analysis

The results clearly demonstrate that the occurrence of unintended consequences depends on the relations involved more than on the ontologies where they are found. It is furthermore apparent that the relations from the OBO relation ontology [19] are less problematic than relations that are less well-founded. While the RO contains OBO style relations (concept/term relations rather than logical relations), RO relations are provided with definitions following the logical pattern A *rel*_*class *_B → all instances of A *rel*_*instance *_some instance of B. This pattern (the all-some pattern) for relationship definition lends itself naturally to the OWL existential restriction translation.

Subtle nuances in meaning are captured in ontology building by the use of different relations. In the case of the relations which we have observed to show high occurrence of unintended consequences, what is going wrong in most cases is that the intended meaning of the asserted relation is not being preserved by the conversion to the logic-based OWL existential restriction. This is most symptomatic for the relation **lacks**, where it is obvious that what is intended to be expressed is precisely the antithesis of existential dependence. The **start **and **end **relations express complicated information about the development of anatomical entities as separately distinguishable entities in their own right, and yet the stages of development which form the range of these relations are not themselves always ontologically necessary for the anatomical entities to exist. The relationships in the chemical structural relationships group are intended by domain modelers to convey information about the structure (constitution) of chemicals rather than their existential dependencies [21].

In the remainder of this section we will expand on and discuss these common sources of unintended consequences and propose solutions for appropriate and intuitive modeling approaches more readily understood by editors as well as users, while still allowing the increased accuracy of the logic-based OWL modeling approach to be preserved.

### Types of unintended consequences and possible corrections

#### Inverted part-of statements

It has been repeatedly emphasized that the expression

*A *subClassOf **part**_**of **some *B*

is not equivalent to

*B *subClassOf **has**_**part **some *A*

[19,22], because the first one makes a claim for all members of the class *A*, whereas the second makes a claim for all members of *B*. This confers a risk of confusion, such as in:

*Interkinetic nuclear migration *SubClassOf

**part**_**of **some *Cell proliferation in forebrain*

The ontological dependence expressed by this assertion is that there are no interkinetic nuclear migration processes without a corresponding cell proliferation in forebrain process. This is obviously false, since interkinetic nuclear migration is a very fundamental cell process, which is not limited to forebrains. An easy fix to this error is the inversion of the expression by using the inverse relationship:

*Cell proliferation in forebrain *subclassOf

**has**_**part **some *Interkinetic nuclear migration*

#### Relations describing chemical structure

ChEBI is richly annotated with relationships between molecular entities, some of which stem from IUPAC. Relationships such as **is_tautomer_of, is_enantiomer_of, is_conjugate_base_of **and **is_conjugate_acid_of, has_functional_parent **and **has_parent_hydride**, express connections between types of molecular entity, typically represented as chemical graphs [21]. Let us analyze the following relational statement:

*Nitrosobenzene *subClassOf

**has_parent_hydride **some *Benzene*

The intended meaning is that the structure of nitrosobenzene molecules derives from the structure of benzene molecules, i.e. by substituting one H of a benzene molecule with an N = O group. This statement makes little sense if applied to all single nitrosobenzene molecules, because the existences of nitrosobenzene molecules do not depend on the existence of a separate benzene molecule, nor are they necessarily physically derived from a preexisting benzene molecule.

There are several approaches to logically describing the relationship between chemical structures while avoiding unintended consequences [21].

One possibility is to make a distinction between material chemical entities (the "real" molecules) and chemical graphs as information artifacts. However, ChEBI's editorial guidelines specify the interpretation of their representational units as classes of molecules and not as information entities, and therefore favours an interpretation of the semantics of the relationships used in terms of the molecules themselves. Another possibility is the use of value restrictions instead of existential restrictions:

*Nitrosobenzene *subclassOf

**has_parent_hydride **only *Benzene*

What is expressed here is that if we compare a nitrosobenzene molecule with other molecules, then we can assert a **has_parent_hydride **relationship only to a molecule of the type benzene. This formulation, without an existential restriction, avoids asserting any ontological dependence between the classes. This means that we no longer require some benzene molecule for each and every nitrosobenzene molecule and so we do not create an unintended consequence. We admit, however, that this solution is suboptimal as it still involves asserting a relationship between two molecules that neither interact nor derive from one another.

#### Reference to missing entities

This has been addressed by Ceusters et al. [23] and Hoehndorf et al. [24], the former advocating the lacks relation, used e.g. in the Protein Ontology, as in the following example:

*Chordin isoform 1 unmodified form *subclassOf

*Chordin isoform 1 *and **lacks_modification**

some *Post-translational protein modification*

The problem is obvious: the existential quantifier asserts the existence of some instance of *Post-translational protein modification *for each and every instance of the *class Chordin isoform 1 unmodified form*, while the intended meaning of **lacks_modification **is exactly the opposite.

For references to missing entities, Ceusters et al. suggest a family of **lacks**_* relations, which relate particulars with universals [23]. This is difficult to express in description logics, as here relations only range over individuals, as explained above. Hoehndorf et al. propose a design pattern for the lacks relation compatible with description logics [25]. However, the proper interpretation of "lacks" relations depends on implicit assumptions which may differ from case to case. If we want to define the class of all mice that lack a tail we can simply negate a parthood assertion:

not (**has**_**part **some *Tail*),

because mice have only one tail. However, if we want to define the class of mice that lack a limb then we do not assume that these mice have no limbs at all. Rather, we should say that they have at most three limbs. In our above example the situation is different because the point is not the lacking of material parts but lacking of participation in a process. This could be expressed as follows:

*Chordin isoform 1 unmodified form *subclassOf

*Chordin isoform 1 *and

not (**participant_of **some

*Post-translational protein modification*)

#### Roles and realizable entities

Roles exist in virtue of a kind of participation of an entity in some process under specific circumstances, during which the entity is said to be playing the role [26]. The assignment of roles is important for the classification of devices or chemical products, e.g. regarding their therapeutic use. A statement such as

*Anisotropine methylbromide *subclassOf

**has_role **some *Anti-ulcer drug*

in ChEBI asserts that each and every anisotropine methylbromide molecule has the role of an anti-ulcer drug. However, this role may never be realized for a particular molecule instance, since that molecule may play a different role in the treatment of a different disease, or play no role at all. It is thus problematic to assert an existential dependence between the molecule and the realization of the role (in the treatment of an ulcer).

A possible solution to this problem is, again, the use of value restrictions to avoid existential dependence [27].

*Anisotropine methylbromide *subClassOf

**has_role **only *Anti-ulcer drug*

Our model needs to accommodate multiple possible drug roles for a given molecule though, since many molecules can be used in different treatment contexts, and even simultaneously. We could express this using disjunction as

*Gemcitabine hydrochloride *subclassOf

**has_role **only

(*Antiviral drug *or *Antineoplastic drug*)

This is problematic as it involves a closed world-like statement (we have to know ALL the possible drug roles that the molecule can play in advance to model this correctly). The usual OWL formalism is based on open world semantics precisely so that we can express incomplete knowledge without creating contradictions as new information becomes available.

Other realizable entities, such as functions, behave similarly [28]. This is particularly apparent in the Gene Ontology molecular function ontology. For example, the statement

*tRNA sulfurtransferase *subClassOf

**has_input **some *Transfer RNA*

asserts a dependency of every instance of *tRNA sulfurtransferase *on some instance of *Transfer RNA*. Functions include the possibility that the bearer of a function is never involved in any process that realizes the function, thus may never have input molecules. This kind of error predominates in the Cross Product sample, especially in the cross product 'GO Molecular Function X ChEBI'. Interrater agreement was low here because of two conflicting positions: (1) the assertion is false, because functions can remain unrealized, or (2) the assertion is true, but the categorization as a function is false, as implied by the suffix "activity". We can avoid the existential implication by using the following model:

*tRNA sulfurtransferase *subClassOf

**has_realization **only

(**has_input **some *Transfer RNA*)

bearing in mind that again, multiple target classes need to be linked by a disjunctive statement.

#### Time dependencies

These are commonly expressed in ontologies encoding development or other time-dependent processes. Kinds of participation in such time dependent processes can be difficult to pin down as can the exact ontological dependence between the process and the material entities. The **start **and **end **relations are intending to express just such time dependencies to do with the development of anatomical structures.

*Pharyngeal endoderm *subClassOf

**end **some

Pharyngula:Prim-15 Roof plate rhombomere 5

subClassOf

**start **some *Segmentation:10-13 somites*

However, the stages of development mentioned may not be complete before the material entity comes fully into existence. They also may not be complete when the material entity stops existing. It is difficult to claim a processual entity (which extends over time) is ontologically necessary for a material entity to exist (the claim of existential dependence) unless the material entity was a clear output of this process. The solution here is, again, to substitute existential restriction by value restriction, such as

*Pharyngeal endoderm *subClassOf

**end **only *Pharyngula:Prim-15*

#### Comparison with extended OBO-OWL translation rules

The conversion from the OBO flatfile format to the OWL representation is a crucial element in our analysis. The proposed OBO Relation Ontology contains definitions for many of the analyzed relations (http://obo.cvs.sourceforge.net/*checkout*/obo/obo/ontology/OBO_REL/ro_proposed.obo). We checked the available conversion software OBO2OWL (http://code.google.com/p/oboformat) and a solution proposed by Hoehndorf et al. [25] for their potential to reduce the error rates of our study. The results are displayed in Additional file 3. As clearly shown, only about 24 erroneous existential restrictions or 2% of the total OBO sample and one erroneous existential restriction (0.1%) of the OBO crossproducts sample would have been translated to a statement without existential dependency. The patterns proposed by [25] for the relations **realized_by **and **lacks_part **have already been discussed above:

*X ***realized_by **only *Y*)

*X *not (**has_part **some *Y *)

The conversion software OBO2OWL maps relational OBO statements to corresponding OWL restrictions with existential quantification only [29].

### Summary of recommendations and usability issues

Having analyzed a sample of OBO ontologies and cross-products for erroneous assertions, we have identified several common patterns leading to unintended consequences and proposed alternative modeling strategies to address these. Several issues of ontology usability have become evident insofar as they have negatively affected the process and the outcome of our rating work. Such issues also impair the use of these ontologies for annotation and complicate their interoperability.

A major deteriorating factor is the proliferation of relations whose exact meaning can often only be vaguely derived from the context of their usage. Our sampling strategy was based on relations, and as such it became evident that a large number of relations are highly ontology-specific. This may make sense in cases where domain-specific requirements lead to the creation of domain-specific relationships, but in many cases (consider the "start" and "end" relationships, for example), the problem did not appear to be motivated by domain restrictions, but rather, we hypothesise that factors of historical contingency and separate development are the dominant causes for the current status.

Another factor in the difficulty in interpretation is the divergence of meaning associated with some terms across ontologies. In particular, the notion of quality, role, or function diverges. This problem is exacerbated by the absence of a clear categorization, committed to an upper level ontology. Divergence is also evident in the distinction between function and process in GO (with all molecular function terms containing the suffix "activity" which rather implies processual entities), which caused disagreement between the raters. This explains statements such as

S-methylmethionine transmembrane

*transporter activity *subClassOf

**part_of **some (S-*methylmethionine transport*

or *Protein kinase activator activity*

subClassOf **positively_regulates **some

*Protein kinase activity*)

Whether or not to reject such axioms fundamentally depends on whether we are guided rather by the intuitive, potentially ambiguous meaning conveyed by the class names, or by upper-level constraints (which would not allow a function to be part of a process).

Such constraints are, however, not yet sufficiently provided by the upper-level ontologies recommended by the OBO foundry, such as the Basic Formal Ontology (BFO) and the OBO relation ontology. This would be a major desideratum for the future, together with a better anchoring of OBO Foundry ontologies into such an upper level.

## Conclusions

Our scrutiny of the OBO Foundry candidate ontologies and cross products yielded a relatively high proportion of inappropriate usages of simple logical constructors. Only focusing on the proper use of existential restriction in class definitions, we found up to 23% of unintended consequences in these constructions. Many Foundry ontologies are widely used throughout the biomedical domain, and therefore such a high error rate seems surprising.

We hypothesize that the main and only reason why this has little affected the usefulness of these ontologies up to now is due to their predominant use as controlled vocabularies rather than as computable ontologies. Misinterpretations of this sort can cause unforeseeable side effects once these ontologies are used for machine reasoning, and the use of logic-based reasoning based on biomedical ontologies is increasing with the advent of intelligent tools surrounding the adoption of the OWL language. It is beyond the scope of this paper to fully evaluate the different benefits and weaknesses of the formal approach vs. the terminological approach, but it should be noted that the trend overall throughout the bio-ontology community is towards greater formalism to address increasingly sophisticated challenges. Therefore, we propose alternative modeling patterns which yield adequate entailments.

Another problem that hindered our experiments is the unclear ontological commitment of many classes and relations in OBO ontologies, which makes it nearly impossible to reach consensus about the truth-value of many of their axioms. This involves not only ambiguities in ontological interpretation of the classes included in the ontologies but also the proliferation of relations which were poorly defined. To address this shortcoming, ontologies can rely on more expressive languages and axiom systems in which the intended semantics of the relations used are constrained, as is done for the OBO relation ontology.

In general, we recommend the OBO community to more actively engage in quality issues in order to make their ontologies fit for use in intelligent systems. This requires a deeper understanding of the underlying formalisms by the ontology developers and curators, an increased awareness by the user community, the development of best-practice guidelines and an auditing strategy, and last but not least, a stricter anchoring in an expressive upper-level ontology and shared relation ontology to help avoid indefinite ontological commitments and ambiguous interpretations.

## Competing interests

The authors declare that they have no competing interests.

## Authors' contributions

SS elaborated the project idea and developed the work plan. IT performed the sampling, the data collection and analysis. MB performed the statistics, elaborated the data presentation, and lead-managed the final manuscript. JH, SS, DS, and MB did the ratings and contributed to the manuscript. All authors have read and approved the final version of the manuscript.

## Supplementary Material

Additional file 1 **Ratings and estimates by relation**. Ratings and estimates for ontologies (top) and cross products (bottom), by relation. Click here for file

Additional file 2 **Ratings and estimates by source**. Ratings and estimates for ontologies (top) and cross products (bottom), by source. Click here for file

Additional file 3 **Axioms with OBO relation ontology and OBO relation ontology candidate relations in the OBO and OBO crossproducts samples**. Only 25 axioms with the relations realized_by and lacks_part would be converted to OWL axioms without unintended existential quantification in the investigated samples according to a proposal for an enhanced mapping of the flatfile OBO format to OWL [25]. † http://www.obofoundry.org/ro/ro.owl; 21 relation types (without obsolete relation types) ‡ http://obo.cvs.sourceforge.net/*checkout*/obo/obo/ontology/OBO_REL/ro_proposed.obo; 151 relation types *: OBO sample with 63 relation types and 1186 axioms investigated; XP sample with 102 relation types and 1650 axioms **: Number of OBO relation ontology resp. OBO relation ontology candidate relation types found in the specific sample ***: Absolute number of investigated axioms with the indicated relation types in the sample (percentage of total sample). Click here for file
