# Supplementary figures and images for: Unintended consequences of existential quantifications in biomedical ontologies
Source: BMC Bioinformatics. 2011 Nov 24;12:456. doi: 10.1186/1471-2105-12-456 (PMC3280341; doi:10.1186/1471-2105-12-456)

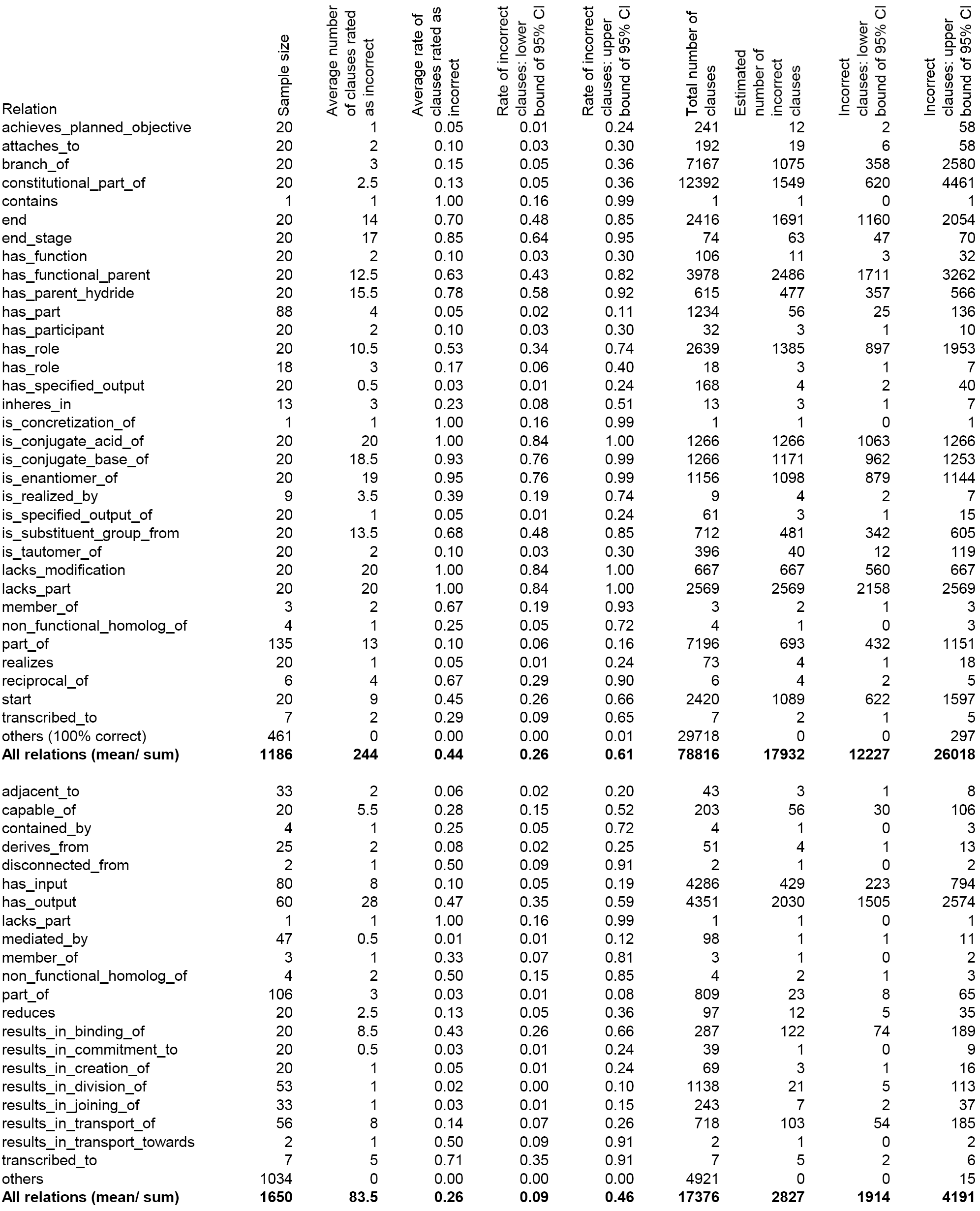

Supplement: Additional file 1 — Ratings and estimates by relation. Ratings and estimates for ontologies (top) and cross products (bottom), by relation. [file 1471-2105-12-456-S1.PNG]

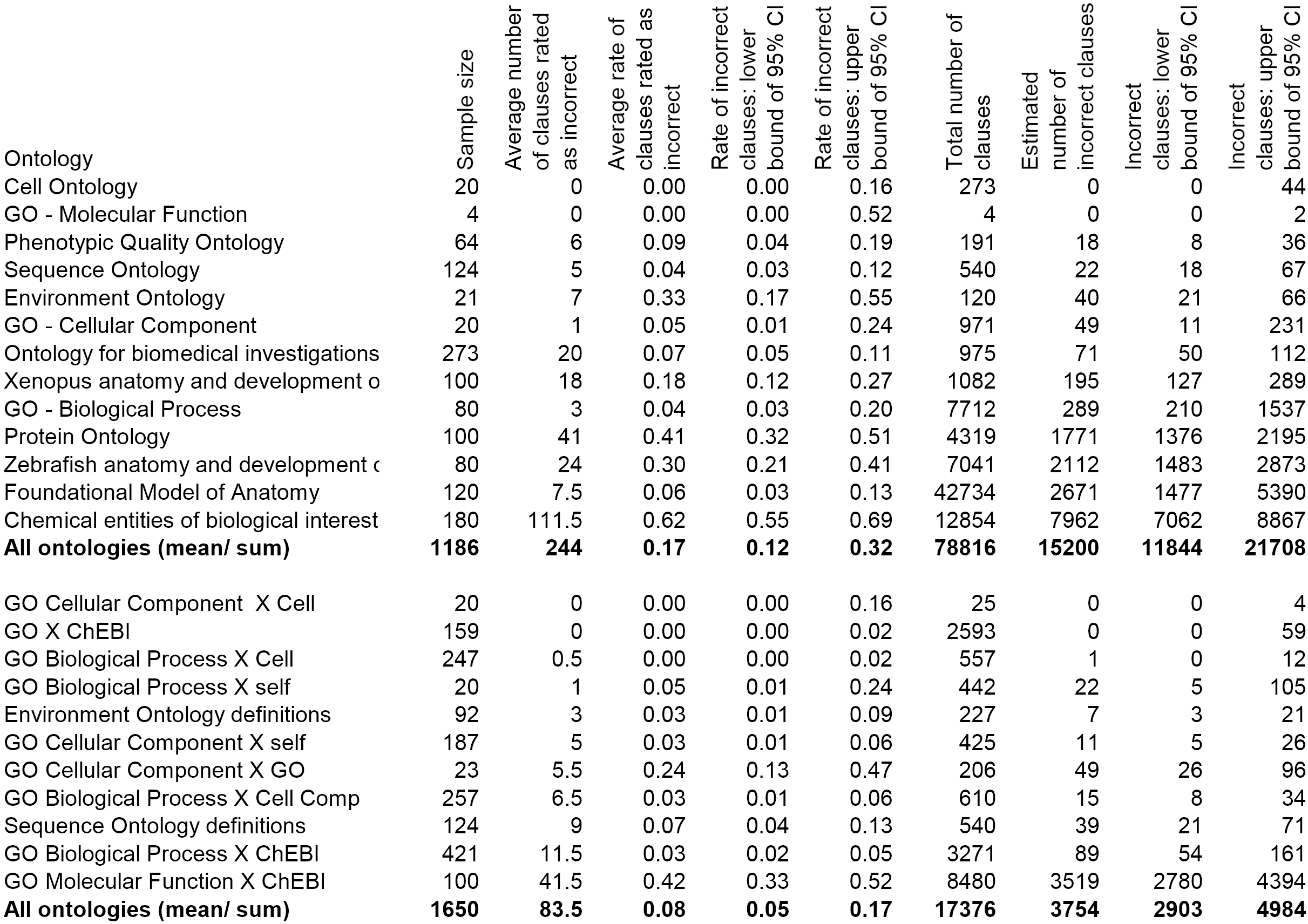

Supplement: Additional file 2 — Ratings and estimates by source. Ratings and estimates for ontologies (top) and cross products (bottom), by source. [file 1471-2105-12-456-S2.PNG]

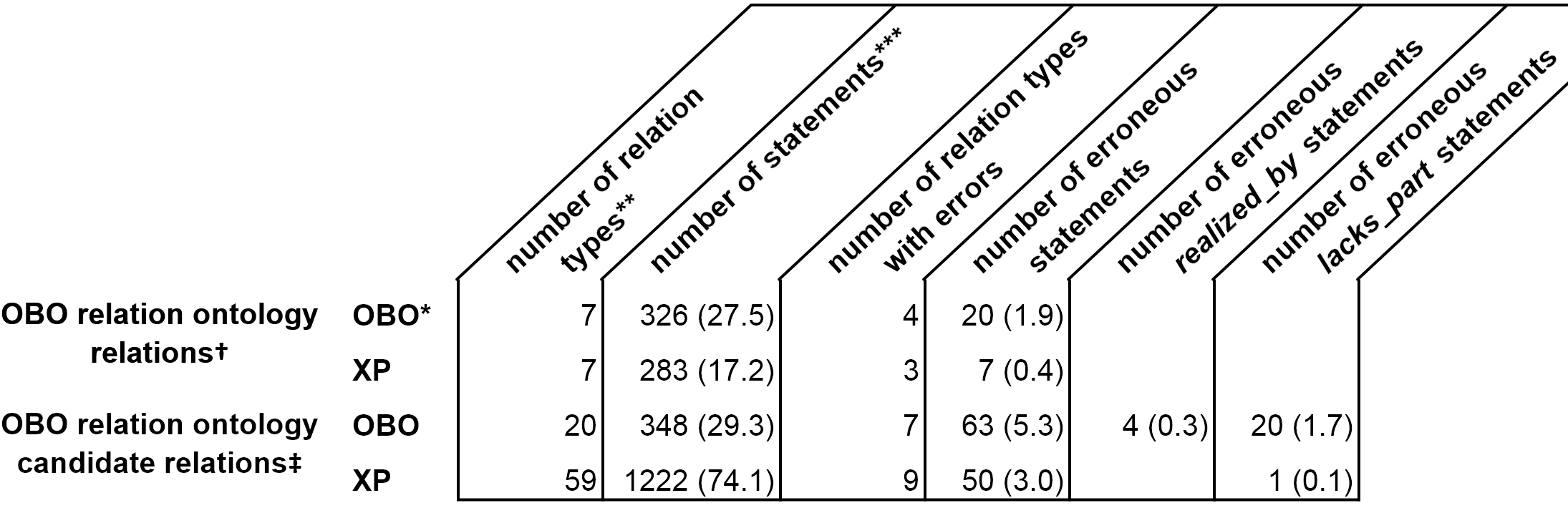

Supplement: Additional file 3 — Axioms with OBO relation ontology and OBO relation ontology candidate relations in the OBO and OBO crossproducts samples. Only 25 axioms with the relations realized_by and lacks_part would be converted to OWL axioms without unintended existential quantification in the investigated samples according to a proposal for an enhanced mapping of the flatfile OBO format to OWL [25]. † http://www.obofoundry.org/ro/ro.owl; 21 relation types (without obsolete relation types) ‡ http://obo.cvs.sourceforge.net/*checkout*/obo/obo/ontology/OBO_REL/ro_proposed.obo; 151 relation types *: OBO sample with 63 relation types and 1186 axioms investigated; XP sample with 102 relation types and 1650 axioms **: Number of OBO relation ontology resp. OBO relation ontology candidate relation types found in the specific sample ***: Absolute number of investigated axioms with the indicated relation types in the sample (percentage of total sample). [file 1471-2105-12-456-S3.PNG]
